# Smaller capillaries improve the small-angle X-ray scattering signal and sample consumption for biomacromolecular solutions

**DOI:** 10.1107/S1600577518007907

**Published:** 2018-06-26

**Authors:** Martin A. Schroer, Clement E. Blanchet, Andrey Yu. Gruzinov, Melissa A. Gräwert, Martha E. Brennich, Nelly R. Hajizadeh, Cy M. Jeffries, Dmitri I. Svergun

**Affiliations:** a European Molecular Biology Laboratory (EMBL), Hamburg Outstation c/o DESY, Notkestrasse 85, 22607 Hamburg, Germany; b European Molecular Biology Laboratory (EMBL), Grenoble Outstation, 71 Avenue des Martyrs, 38042 Grenoble, France

**Keywords:** small-angle X-ray scattering, protein solution, radiation damage, flow through sample environment

## Abstract

Radiation damage by intense X-ray beams at modern synchrotron facilities is one of the major complications for biological small-angle X-ray scattering (SAXS) investigations of macromolecules in solution. To limit the damage, samples are typically measured under a laminar flow through a cell (typically a capillary) such that fresh solution is continuously exposed to the beam during measurement. The diameter of the capillary that optimizes the scattering-to-absorption ratio at a given X-ray wavelength can be calculated *a priori* based on fundamental physical properties. However, these well established scattering and absorption principles do not take into account the radiation susceptibility of the sample or the often very limited amounts of precious biological material available for an experiment. Here it is shown that, for biological solution SAXS, capillaries with smaller diameters than those calculated from simple scattering/absorption criteria allow for a better utilization of the available volumes of radiation-sensitive samples. This is demonstrated by comparing two capillary diameters *d*_i_ (*d*_i_ = 1.7 mm, close to optimal for 10 keV; and *d*_i_ = 0.9 mm, which is nominally sub-optimal) applied to study different protein solutions at various flow rates. The use of the smaller capillaries ultimately allows one to collect higher-quality SAXS data from the limited amounts of purified biological macromolecules.

## Abstract 2

Sample exposure cells of small-angle X-ray scattering instruments often utilize cylindrical capillaries where the diameter, or path length, is typically selected to balance between scattering and absorption. Here it is demonstrated that, for radiation-sensitive solution samples, using capillaries with a diameter smaller than the optimal path length in combination with continuous sample flow improves the quality of the scattering signal for a given quantity of material.

## 1. Introduction   

Small-angle X-ray scattering (SAXS) is used to probe the structure of biological macromolecules in solution (Graewert & Svergun, 2013 ▸; Skou *et al.*, 2014*a* ▸; Svergun & Koch, 2003 ▸; Svergun *et al.*, 2013 ▸; Schroer & Svergun, 2018 ▸). The most advanced studies are conducted at synchrotron SAXS beamlines, partially or fully dedicated to structural biology (Blanchet *et al.*, 2015 ▸; Pernot *et al.*, 2013 ▸; David & Pérez, 2009 ▸; Inoue *et al.*, 2013 ▸; Acerbo *et al.*, 2015 ▸; Classen *et al.*, 2013 ▸; Kirby *et al.*, 2013 ▸; Li *et al.*, 2016 ▸). The SAXS data collection on bio­macromolecular solutions presents specific experimental challenges. The solutes are often very dilute (over 99% water by volume) and have low X-ray contrast resulting in very weak scattering intensities upon subtraction of the solvent scattering. Synchrotron SAXS capitalizes on the brilliance of the X-ray source to improve the signal intensity; however, the high brilliance also has a downside. As the samples are in aqueous solutions, the photolytic production of free radicals and solvated electrons from the solvent by the X-ray beam may cause radiation damage and the formation of macromolecular aggregates that may render SAXS profiles uninterpretable (Garrison, 1987 ▸; Maleknia *et al.*, 2001 ▸; Kuwamoto *et al.*, 2004 ▸). Since the observable effect of radiation damage depends not simply on the dose deposited on the sample but largely on the tendency of the solute to aggregate, it is not possible to adequately predict the magnitude of the expected effect prior to the measurement. Complicating matters, the sample itself may only be available in limited quantities. Therefore, there is a need to balance SAXS data collection between the effects of potential radiation damage and sample consumption to maximize the data quality, *i.e.* optimize the signal-to-noise ratios in the recorded weak scattering intensities from un­damaged samples. Mitigating the effects of radiation damage is particularly important for biological SAXS (bioSAXS) measurements (Brooks-Bartlett *et al.*, 2017 ▸; Hopkins & Thorne, 2016 ▸; Jeffries *et al.*, 2015 ▸) and, in order to reduce this effect, several schemes can be employed. These include continuous sample flow, beam attenuation, the addition of small ‘scavenger’ molecules or cryo-cooling (Fischetti *et al.*, 2003 ▸; Jeffries *et al.*, 2015 ▸, 2016 ▸; Kirby *et al.*, 2016 ▸; Kuwamoto *et al.*, 2004 ▸; Meisburger *et al.*, 2013 ▸, Sahle *et al.*, 2015 ▸). In the following, we show that the geometry of the sample exposure cell can also be optimized to limit the impact of radiation damage and improve the measured SAXS profiles from radiation-sensitive samples.

The magnitude of the SAXS intensity, *I*, measured from a dilute protein solution can be described as
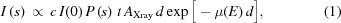
where *s* is the wavevector momentum transfer that relates to the X-ray wavelength λ and the scattering angle 2θ as *s* = 4πsinθ/λ. Here, *c* denotes the solute concentration, *I*(0) the forward scattering, *P*(*s*) the normalized form factor [*i.e.* *P*(0) = 1], *t* the exposure time, 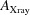 the X-ray beam-area at the sample position, *d* the sample path length and μ(*E*) the total linear attenuation coefficient. Intuitively, reducing the time in the X-ray beam decreases *I*(*s*); however, measuring samples as a set of sequential short exposures, as opposed to one long exposure, is useful to detect the onset of radiation damage and ensure that only the data collected from the sample unaffected by beam-induced changes are averaged, thereby improving the data accuracy. The total linear attenuation coefficient is dependent on the X-ray absorption and scattering cross sections at a given X-ray photon energy *E*. Equation (1) indicates that the strongest scattering signal at a fixed concentration is given for the sample path length *d*_opt_ = 1/μ (see §S1, Fig. S1, of the supporting information), which is typically met in solution SAXS measurements. In practice, cylindrical glass capillaries with diameters between 1 and 2 mm are used, as these match the optimum sample path length of *d*_opt_ = 1.0 mm (at *E* = 8 keV) and *d*_opt_ = 1.9 mm (at *E* = 10 keV) for water, which is a reasonable approximation for diluted aqueous macromolecular samples.

This calculation of the optimal path length that maximizes the scattering signal only takes into account the stochastic X-ray interaction with the sample as described by μ(*E*) without accounting for the effect of radiation damage. The latter may lead to the formation of diverse solute states, mostly aggregates in this study, which all yield different form factors *P*(*s*). It is from *P*(*s*) that structural information can be obtained and, ultimately, a successful bioSAXS experiment needs to maximize *I*(*s*) *without* incurring X-ray induced changes in *P*(*s*) during the X-ray exposure.

Since macromolecular samples have limited in-beam lifetimes and are often available in limited quantities, it is necessary to optimize the SAXS signal that can be collected as a function of the sample volume while minimizing radiation damage. The diameter of the cylindrical capillary influences the intensity *I*(*s*), the absorption and therefore defines how efficiently the sample volume is used. The beam area at many SAXS beamlines is in the range 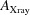 = 100–500 µm × 100–500 µm and consequently a large quantity of the sample, if housed within a 1–2 mm capillary, is not exposed at all and exits the capillary unmeasured. Capillaries with smaller diameters increase the ratio of illuminated to non-illuminated volume, thus improving the sample utilization. Moreover, for continuous-flow experiments, smaller capillaries enable higher flow speeds refreshing the sample through the beam faster and thus reducing the cumulative effects of radiation damage. Therefore, although smaller-diameter capillaries based on first principles described in equation (1) lower the scattering intensity, the decrease in path length may be outweighed by a more efficient data collection scheme to ultimately yield higher-quality scattering data from undamaged samples. In this work we present comparative studies demonstrating that, indeed, capillaries with diameters smaller than the calculated optimum thickness do provide overall improvements to the SAXS profiles measured from radiation-sensitive biological samples.

## 2. Experimental details   

### 2.1. Sample preparation   

Powdered ultrapure chicken egg-white lysozyme was purchased from Affymetrix USB. Bovine serum albumin (BSA; lyophilized powder, crystallized, >98%) was purchased from Sigma. In all instances the protein samples were freshly prepared at room temperature (*T* ≃ 20°C), just prior to the SAXS measurements. Individual aliquots of the respective protein powders were separately dissolved in 50 ml of 40 m*M* KH_2_PO_4_, 60 m*M* KCl, pH 6.8 [this phosphate buffer was chosen to increase the susceptibility of both proteins to radiation damage (Jeffries *et al.*, 2016 ▸)]. The solutes were 0.22 µm filtered to produce lysozyme and BSA samples at a final protein concentration of *c* = 4.4 mg ml^−1^ and *c* = 2.9 mg ml^−1^, respectively. The final concentration was evaluated by UV absorption using a NanoDrop spectrophotometer. The *A*_280nm_ absorption coefficients expressed as *E*_0.1%_ (= 1 mg ml^−1^) were calculated from the primary amino acid sequence of each protein using *ProtParam* (Gasteiger *et al.*, 2005 ▸: BSA *E*_0.1%_ = 0.614; lysozyme *E*_0.1%_ = 2.653). The same phosphate buffer used to prepare the protein samples was also employed as the matched solvent blank for the subtraction of the background scattering.

### 2.2. SAXS data collection   

SAXS measurements were performed at the EMBL P12 bioSAXS beamline, EMBL/PETRA III, Hamburg, Germany (Blanchet *et al.*, 2015 ▸; Hajizadeh *et al.*, 2018 ▸), using an X-ray photon energy of *E* = 10 keV (λ = 0.124 nm) with a beam size of 200 µm × 350 µm (V × H, full width at half-maximum, FWHM) and a flux of *F* = 5 × 10^12^ photons s^−1^ at the sample position. Standard batch mode measurements were performed using the robotic P12 sample changer (Blanchet *et al.*, 2015 ▸; Round *et al.*, 2015 ▸) both with and without continuous in-capillary sample flow through the beamline (horizontally oriented under vacuum). Before and after the protein solutions the corresponding buffer solutions were measured. Between each measurement the capillaries were extensively cleaned to remove any deposits due to radiation-induced aggregates.

Two types of quartz glass capillaries with different diameter were used: (1) a capillary with an outer diameter of *d*_o_ *=* 1.8 mm and inner diameter of *d*_i_ *=* 1.7 mm, close to the optimum path length (*d*_opt_ = 1.9 mm) for 10 keV X-rays, and (2) a capillary with *d*_o_ = 1.00 mm and *d*_i_ = 0.91 mm. Both capillaries were purchased from Hilgenberg, Germany. In the following, they will be referred to as the *d*_i_ = 1.7 mm and *d*_i_ = 0.9 mm capillaries, respectively. For all measurements, a constant sample volume of *V*_tot_ = 20 µl was loaded into the capillaries that were maintained at a constant temperature of *T* = 10°C.

SAXS data were recorded using a PILATUS 2M photon-counting detector (DECTRIS, Switzerland) from samples and buffers using exactly the same conditions at several different flow rates and total exposure times of the solutions as described in the *Results* [Sec sec3]. For all experiments, two-dimensional (2D) SAXS data were recorded as a sequential set of images (frames) for every 50 ms of exposure time (45 ms collection time + 5 ms detector readout time) during the course of X-ray exposure.

### 2.3. SAXS data analysis   

The 2D images on the detector were used to estimate the number of photons scattered by the proteins in each sample. First, the scattering patterns for the samples and corresponding buffers were scaled to the storage ring current to take into account small variations in the intensity of the incident X-rays. Then the data collected from the buffer were subtracted from that of the respective sample to obtain subtracted 2D images of the proteins in solution. For each subtracted 2D image, the number of photons counted by the pixels in the range 0.12 nm^−1^ < *s* < 2.9 nm^−1^ were summed to obtain the integrated scattered photon count. The summation was conducted until the frames showed radiation damage yielding the integrated photon count, *I*_int_, from the undamaged macromolecules in the sample.

To assess the time point of the onset of radiation damage and to obtain 1D-reduced scattering profiles for subsequent analysis, the unsubtracted 2D images underwent data reduction using the P12 beamline SASFLOW pipeline (Franke *et al.*, 2012 ▸). Briefly, the 2D SAXS patterns were normalized to the transmitted beam and azimuthally averaged. The resulting SAXS curves were then analysed using the correlation map method (*CorMap*; Franke *et al.*, 2015 ▸) whereby the one-dimensional (1D) profiles from the sequentially acquired images were pairwise compared for statistical similarity across the entire *s*-range (relative to the first data frame). Only the frames above a similarity significance threshold of α ≥ 0.01 were used to generate the (frame-)averaged SAXS profile for the given sample and buffer. The averaged protein SAXS data were corrected for the averaged buffer/background scattering, measured under the exact same conditions, to yield the SAXS signal from the protein, 〈*I*(*s*)〉, without radiation damage. In the pure buffer solutions studied no radiation damage was observed across the individual data frames as assessed using *CorMap*.

In addition to the *CorMap* pairwise evaluation of each data frame, all individual images measured from a sample underwent data reduction and buffer subtraction using the averaged buffer data to produce a set of single SAXS curves *I*(*s*) from which the consistency of the radius of gyration *R*_g_ across frames was evaluated using the Guinier approximation [*I*(*s*) 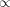 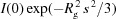, where *I*(0) denotes the extrapolated forward scattering at zero angle (Guinier, 1939 ▸)].

While *CorMap* assesses systematic (correlated) +1 or −1 variances (co-variances) in the intensities between data frames across the entire data range, the *R*_g_ assessment from the Guinier approximation uses only intensities at the very low angles calculated from a linear fit to the data [when expressed as ln*I*(*s*) *versus* *s* ^2^, to an *R*_g_ *s* ^max^ = 1.3]. The linear fit from which *R*_g_ is calculated from the Guinier plot (proportional to the negative slope) is affected by both signal-to-noise and the associated errors on the data. As a result, the *R*_g_ error estimates may be relatively large, especially when using a single data frame. On comparing the data frames, the *R*_g_ approach does not take into account systematic discrepancies that may occur during the linear fitting (linear correlation coefficients are insufficient to detect such discrepancies). Therefore, the joint use of *CorMap* and the Guinier *R*_g_ provides a reliable estimate of the onset of damage. *CorMap* also has the advantage of assessing whether systematic discrepancies between the compared intensities occur outside of the Guinier region (covering the cases when the effects of damage manifest themselves at higher angles, *i.e.* at shorter length scales).

As the main aim of averaging the single SAXS curves unaffected by radiation damage is to reduce the noise in the data, it is essential to assess the effect of changing the capillary diameter on the resulting noise level. Therefore, the 〈*I*(*s*)〉 curves were further processed with the program *AUTOGNOM* from the *ATSAS* program package (Petoukhov *et al.*, 2012 ▸; Franke *et al.*, 2017 ▸) to obtain the smoothed reciprocal-space fit to the experimental, undamaged data, *I*_AG_(*s*) (from the calculated probable real-space distance distribution). In all instances, reciprocal-space fit *CorMap* *p*-values were greater than 0.01, indicating that no systematic deviations are present between the calculated fit and the experimental data (Franke *et al.*, 2015 ▸). The normalized deviation Δ〈*I*(*s*)〉 of the experimental SAXS curves, 〈*I*(*s*)〉, from the smoothed *AUTOGNOM* *I*_AG_(*s*) function was computed for each sample such that 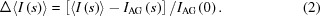
From this normalized difference curve, a scalar quantity DEV was determined as
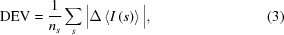

*i.e.* the total sum of the modulus of the normalized deviations divided by the number of experimental data points *n*_*s*_. For noisy data, it is expected that the fluctuations with respect to the smooth curve *I*_AG_(*s*) will be high, yielding larger DEV, while lower DEV values point to lower relative noise levels. For each protein species the same respective *s*-range and a similar number of data points *n*_*s*_ were used to compute the DEV value.

In order to determine the quality and information content in the SAXS curves from which structural information for modelling can be determined, the radially averaged data were further analysed with the *ATSAS* program *SHANUM* (Konarev & Svergun, 2015 ▸). With this approach, the numbers of reliably detectable Shannon channels *M*_S_ and therefore the maximum momentum transfer in the useful data range *s*_opt_ were determined for both capillaries.

## 3. Results   

### 3.1. Sample geometry and effective sample exposure volumes   

**Figure 1:**
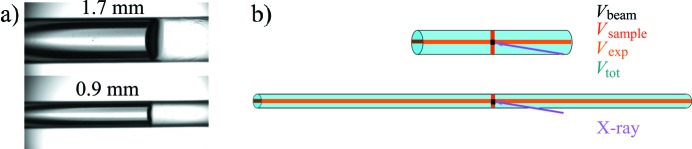
(*a*) Magnified images of the two types of horizontal capillaries placed in the sample changer, loaded with a solution (side view), that can be held fixed in the beam (for stationary SAXS measurements) or can be flowed left to right during X-ray exposure. (*b*) The scheme of the accessible sample volume in the different geometries. Here, black squares mark the position and size of the X-ray beam, and represent the focal volume *V*_beam_ of the sample exposed to the beam; the red rectangles indicate *V*_sample_; the orange rectangles indicate the total volume exposed *V*_exp_ when flowing the sample; the total loading volume *V*_tot_ is shown in blue. The arrows indicate the direction of the X-ray beam propagation. Both capillaries in (*b*) have the same total sample volume *V*_tot_.

Fig. 1(*a*) ▸ shows the images taken from a video camera for online visualization of the two cylindrical sample capillaries with the internal diameters *d*_i_ = 1.7 mm and *d*_i_ = 0.9 mm placed in the evacuated sample exposure unit during the automated sample loading. The video microscope detects the meniscus of the sample and places it such that the X-ray beam fully hits the solution that is subsequently measured either at a fixed position or with the sample flow enabled. In a continuous-flow operation, the flow speed is automatically adjusted such that the full volume of the sample is passed through the incident X-ray beam during the total exposure time. The data are acquired as a set of individual images, typically 20 × 50 ms during the exposure time. In Fig. 1(*a*) ▸ the sample flows horizontally from left to right, with the X-ray beam oriented at 90° to the capillary, progressing through the sample from the bottom of the image to the top, with the horizontal beam dimension parallel to the flow direction and the vertical beam dimension out of the plane of the image. The beam is aligned to hit the centre of the capillary, maximizing the path length through the sample.

We shall distinguish the following volumes inside the capillary: the sample volume needed to fill the capillary, *V*_sample_, which is approximated by the size of the beam in the horizontal direction multiplied by the cross-sectional area of the capillary; the volume of *V*_sample_ actually exposed in the X-ray beam, *V*_beam_, that corresponds to the beam size in both horizontal and vertical directions multiplied by the capillary diameter; and the sample volume exposed when flowing the sample *V*_exp_. Fig. 1(*b*) ▸ shows the geometry schematically. As can be seen, the volume exposed at a given time is noticeably smaller than *V*_sample_. The corresponding volumes consumed but *not* exposed by the beam are, for fixed sample measurements, Δ*V*_loss,fix_ = *V*_sample_ − *V*_beam_, and for the flowing samples Δ*V*_loss,flow_ = *V*_tot_ − *V*_exp_. The ratio between *V*_beam_ and *V*_sample_ (and also of *V*_exp_ and the total sample volume *V*_tot_) reflects the sample consumption efficiency. Table 1 ▸ shows the corresponding values for *d*_i_ = 1.7 mm and *d*_i_ = 0.9 mm capillaries where the effect of reducing the internal diameter is immediately obvious under the flow conditions. Indeed, a significantly higher proportion of the total sample is exposed to the X-ray beam upon reducing the capillary diameter.

For the measurements in the fixed position, the larger capillary needs about 3.5 times more volume for filling compared with the smaller one but only a 1.9 times larger volume is actually exposed to the beam and thus contributes to the SAXS signal. When flowing a total solution volume of *V*_tot_ = 20 µl, for the *d*_i_ = 0.9 mm capillary the exposed volume is even about 1.9 times larger than for *d*_i_ = 1.7 mm. This results in a consumption efficiency of 42% (*d*_i_ = 0.9 mm) in comparison with 22% (*d*_i_ = 1.7 mm).

### 3.2. Data collection without sample flowing   

**Figure 2:**
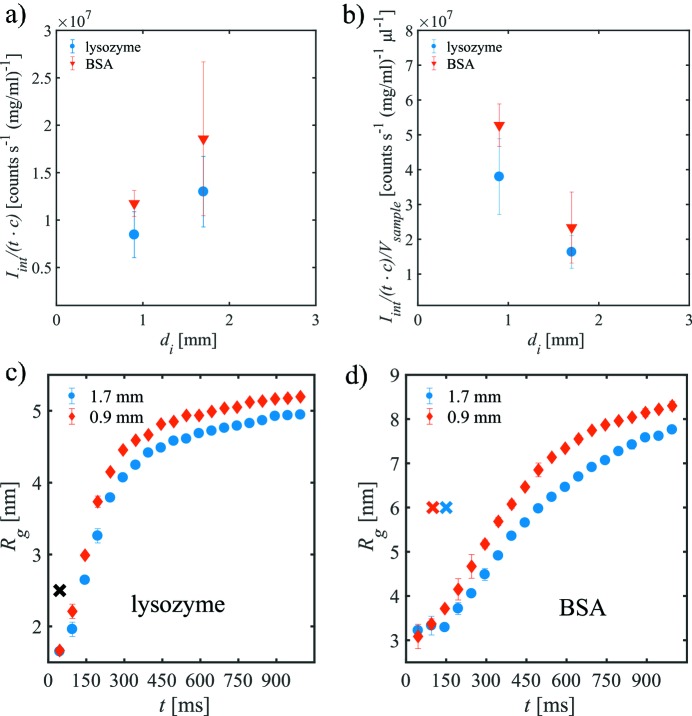
(*a*) Integrated scattering intensity per time *t* and per 1 mg ml^−1^ of lysozyme and BSA concentration *c* in both capillaries. The data were corrected for absorption by the walls of the quartz capillaries. (*b*) Intensity per time and per sample volume, *V*_sample_, needed to fill the capillary, that is much larger than the focal volume of the beam (see Table 1 ▸). Radii of gyration of the fixed protein solution as a function of exposure time for (*c*) lysozyme and (*d*) BSA. The crosses mark the number of single SAXS curves accepted by *CorMap* (blue: *d*_i_ = 1.7 mm; red: *d*_i_ = 0.9 mm; black: for both).

Besides the overall sample consumption, the integrated scattering intensity *I*_int_ from the protein solutions in both capillaries is of major relevance for successful SAXS measurements. For the background-corrected 2D data, the averaged integrated scattering intensity per unit time *t* and per 1 mg ml^−1^ of sample, *I*_int_/(*tc*), has been determined for lysozyme and BSA protein samples (Fig. 2*a* ▸). These are corrected for the absorption of the quartz glass capillaries by dividing through the respective transmissions *T*.

For *d*_i_ = 1.7 mm, the scattering intensity in the fixed position is higher than that for *d*_i_ = 0.9 mm. Thus, as expected from theory, decreasing the capillary diameter leads to a decrease of the integrated scattering per unit time. On the other hand, the scattering intensity per time *and* per volume sample loaded, *V*_sample_, is better for the smaller capillary (Fig. 2*b* ▸). Thus, a higher sample consumption efficiency accompanied by a lower scattering intensity still may yield overall improved scattering intensity statistics if sufficient number of SAXS curves without radiation damage can be measured.

The onset of the radiation damage can be seen by observing the evolution of the radius of gyration *R*_g_ with time. Figs. 2(*c*) and 2(*d*) ▸ show the radius of gyration averaged over several repetitions as a function of exposure time *t* for fixed samples in both capillaries for lysozyme and BSA, respectively. For the lysozyme sample at fixed position (Fig. 2*c* ▸), *R*_g_ increases already after *t* = 45 ms, independently of the capillary diameter. For longer exposure times, the *CorMap* analysis does not find any statistical similarity (black cross). In this situation, no reliable information can be obtained from the data, as the radiation damage is already present in the first SAXS curve. In the case of BSA, which is slightly less radiation sensitive, a similar result is found (Fig. 2*d* ▸).

Summarizing, for the fixed sample, the onset of radiation damage does not depend on the capillary diameter, whereas the total scattering intensity per time *and* per volume sample, *V*_sample_, is higher for the smaller capillary. Thus, the lower sample consumption for the *d*_i_ = 0.9 mm capillary allows for a larger number of repeated measurements which, in turn, would allow one to obtain a larger summed scattering intensity. A natural way of repeating the experiments uses the sample flow within the capillary, and this is discussed in the next section.

### 3.3. Data collection with flowing sample   

#### 3.3.1. Flow rates   

The influence of the capillary size on the measured SAXS intensities from samples under continuous flow through the X-ray beam was studied using a fixed total sample volume *V*_tot_ for various sample delivery volume flow rates *Q* (from *Q* = 10 µl s^−1^ to *Q* = 40 µl s^−1^). The corresponding linear sample flow speed 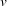 = 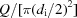, is obviously higher for the *d*_i_ = 0.9 mm capillary compared with the *d*_i_ = 1.7 mm capillary if the samples are delivered at the same flow rate, resulting in shorter average dwell times *t*_dwell_ = *w*/*v* of the protein solution in the X-ray beam of width *w* (in this case, 350 µm) and thus a lower average radiation dose (see §S2 of the supporting information).

The flow properties of liquids in capillaries are usually characterized by the Reynolds number Re = ρ*vd*_i_/η (Rott, 1990 ▸), where ρ denotes the density of the fluid and η the dynamic viscosity. For the solution samples described here (consisting of 99.5% by mass of water) and for the given capillary diameters, the highest Reynolds number is Re = 43 (*T* = 10°C). This value is much lower than the critical value of Re ≃ 2000 (Rott, 1990 ▸) required for the breakdown of laminar flow, and it is thus assumed that, for the dilute protein samples, the laminar flow regime is maintained.

The flow measurements were performed for a total sample volume of *V*_tot_ = 20 µl. Different flow rates through the X-ray beam were obtained by changing the total number of short (*t*_exp_ = 50 ms) sequentially measured SAXS exposures, *N*_SAXS,_ such that
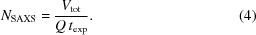
(Note: *t*_exp_ = 50 ms is the sum of collection time *t*_coll_ = 45 ms per curve and a read-out time of *t*_read_ = 5 ms of the Pilatus 2M detector.) The total exposure time, *t*_tot_, of a sample, *i.e.* the maximum illumination of each sample in the X-ray beam (irrespective of radiation damage), is given by *t*_tot_ = *t*_exp_ *N*_SAXS_. The experimental parameters summarized in Table 2 ▸ correspond to the SAXS data collected for the two protein samples in both capillaries at different flow rates using the same total volume.

The laminar flow of protein solutions can be described by a simple parabolic radial velocity profile (Rogers, 1992 ▸). At the capillary walls, the flow velocity approaches zero, and radiation damage is expected to be stronger (Nielsen *et al.*, 2012 ▸). For the given linear flow speeds *v*, the velocity profiles for both capillaries at different flow rates indicate a much thinner boundary layer of slowly moving sample for the *d*_i_ = 0.9 mm capillary, and hence a lower probability of the deposition of protein and aggregates (see §S3, Fig. S2).

#### 3.3.2. Influence of the capillary size on the onset of the radiation damage   

**Figure 3:**
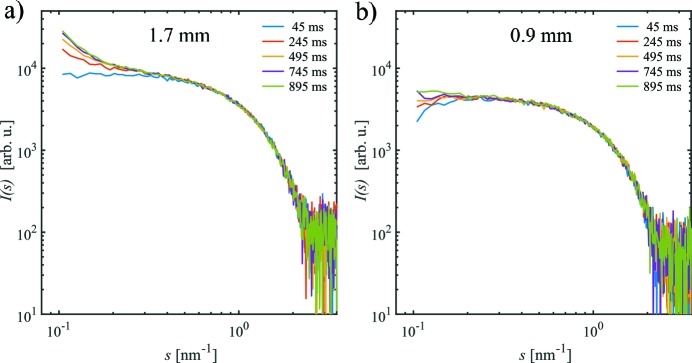
Single SAXS curves *I*(*s*) *versus* *s* for the lysozyme sample in (*a*) *d*_i_ = 1.7 mm and (*b*) *d*_i_ = 0.9 mm capillaries at a total volume flow rate *Q* = 20 µl s^−1^. With increasing exposure time, the scattering intensity at low *s* increases due to the formation of radiation-induced aggregates, especially in the larger-diameter capillary.

Fig. 3 ▸ shows a series of single 1D-SAXS curves of lysozyme at the flow rate *Q* = 20 µl s^−1^ for *d*_i_ = 1.7 mm and *d*_i_ = 0.9 mm capillaries. In both cases, 20 single SAXS profiles were collected using continuous flow. For *d*_i_ = 1.7 mm, the radiation damage is apparent after an exposure of *t* = 245 ms, as is observed by a systematic increase in the scattering intensities near zero angle and an increase in the negative slope at very low values of *s* (*s* < 0.5 nm^−1^, Fig. 3*a* ▸). In comparison, for the *d*_i_ = 0.9 mm capillary, the onset of radiation damage is significantly extended whereby the formation of aggregates is not apparent in the data until *t* = 495 ms (Fig. 3*b* ▸).

**Figure 4:**
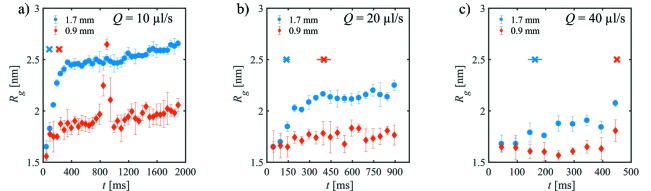
Radius of gyration, *R*_g_, of lysozyme as a function of exposure time *t* at different flow rates for both capillaries: (*a*) for *Q* = 10 µl s^−1^, (*b*) *Q* = 20 µl s^−1^, (*c*) *Q* = 40 µl s^−1^. The crosses mark the number of single SAXS curves accepted by *CorMap* (blue: *d*_i_ = 1.7 mm; red: *d*_i_ = 0.9 mm).

The onset of the radiation damage is reflected yet more clearly in the radius of gyration *R*_g_, and Fig. 4 ▸ shows *R*_g_ as a function of exposure time *t* for different volume flow rates in both capillaries. Flowing the sample at *Q* = 10 µl s^−1^ somewhat reduces the apparent *R*_g_ compared with the fixed sample but only slightly diminishes the radiation damage (Fig. 4*a* ▸). For *d*_i_ = 1.7 mm, after *t* = 90 ms, radiation damage is already detectable, whereas, for *d*_i_ = 0.9 mm, data up to *t* = 200 ms can be averaged. It appears that the size and/or amount of aggregates in the smaller capillary is lower than for the larger capillary, possibly thanks to higher linear flow speed and thus lower radiation dose in the former case. The increase in *R*_g_ at *t* = 800 ms for *d*_i_ = 0.9 mm may be attributed to the formation of larger aggregates similar to those present for *d*_i_ = 1.7 mm, which are, however, quickly removed after *t* = 1000 ms due to the volume flow. This effect could be similar to the vortex-ring flow which circulates the sample solution between the centre and the wall of the capillary as reported previously for capillary flow (Nielsen *et al.*, 2012 ▸).

As expected, less radiation damage is observed when the flow rate increases. Notably, however, the number of radiation-damage-free SAXS curves for *Q* = 20 µl s^−1^ (Fig. 4*b* ▸) and *Q* = 40 µl s^−1^ (Fig. 4*c* ▸) is higher for the *d*_i_ = 0.9 mm capillary than for the *d*_i_ = 1.7 mm. Additionally, for flowing samples, *R*_g_ of the aggregates formed due to radiation damage reaches a plateau value, which decreases continuously with increasing flow rate. Similar results are obtained for BSA (see §S4, Fig. S3), for which the total effect of radiation damage is slightly lower and the solutions are even partially under-exposed, *i.e.* actually more SAXS profiles could be taken before the radiation damage sets in.

#### 3.3.3. Influence of the capillary size and flow rates on the integrated scattering   

When the samples are measured at a continuous flow the smaller-diameter capillary is clearly more effective than the larger capillary in limiting the rate of radiation damage of the radiation-sensitive protein samples. This stems from the higher linear flow speed, *v*, and the associated increase in replenishing the sample through the capillary. As a result, larger numbers of individual SAXS data frames can be collected in succession that, after the frame averaging, improve the signal-to-noise ratios in the resultant SAXS curves. Indeed, the final integrated scattering *I*_int_ collected from the undamaged samples is higher for the *d*_i_ = 0.9 mm capillary compared with the *d*_i_ = 1.7 mm capillary, *i.e.* the reduction in scattering intensity for the 0.9 mm system as predicted by equation (1) (and as discussed for the fixed position results, §3.2) is offset by faster flows reducing the radiation damage combined with a higher proportion of sample volume being illuminated during the total exposure time.

**Figure 5:**
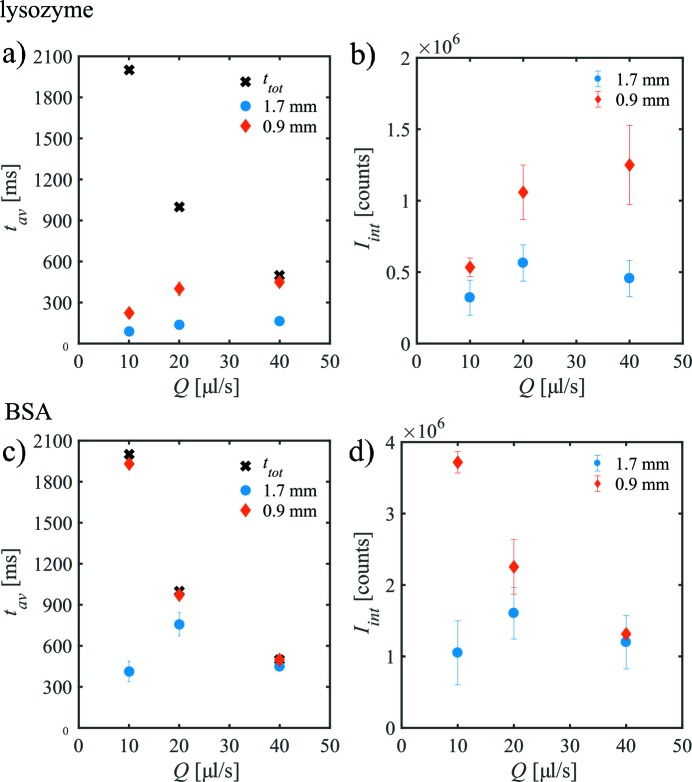
The exposure time before showing radiation damage, *t*_av_, and the integrated scattering intensity *I*_int_, as a function of volume flow rate *Q* for lysozyme (*a*, *b*) and BSA (*c*, *d*) solutions in both capillaries. The total exposure time *t*_tot_ of each measurements is also shown. (Data and error bars are derived from repeated measurements.)

It is instructive to analyse how long the samples can be exposed to the X-ray beam as a function of flow rate before radiation damage sets in, and this can be also easily tested during a SAXS measurement. Fig. 5(*a*) ▸ shows the exposure time, *t*_av_, for which the lysozyme sample can be measured before the onset of radiation damage plotted against the flow rate *Q*. An increase of *t*_av_ with increasing flow rate and, more importantly, a larger exposure time for *d*_i_ = 0.9 mm compared with *d*_i_ = 1.7 mm is observed before radiation damage sets in. The figure also shows the total exposure time *t*_tot_ for the entire set of the SAXS curves. In the case of *d*_i_ = 1.7 mm, radiation damage is present for all flow rates studied; for *d*_i_ = 0.9 mm at *Q* = 40 µl s^−1^, nearly all SAXS curves are similar and can be averaged.

Fig. 5(*b*) ▸ shows the integrated scattering intensity from the SAXS patterns before showing radiation damage, *I*_int_, determined for lysozyme as a function of volume flow rate for both capillaries. Increasing the rate from *Q* = 10 µl s^−1^ to 20 µl s^−1^, the integrated intensity of the intact sample increases as fewer SAXS curves exhibit radiation damage. In the case of *d*_i_ = 1.7 mm, *I*_int_ is the same within the error for *Q* = 20 µl s^−1^ and *Q* = 40 µl s^−1^. A similar result is observed for the smaller capillary.

In the case of BSA, the situation is somewhat different, as the protein is less radiation sensitive (Figs. 5*c*, 5*d* ▸) (Jeffries *et al.*, 2015 ▸). Fig. 5(*d*) ▸ shows *I*_int_ as a function of flow rate. For the larger capillary, the optimum flow condition with the largest *I*_int_ is at *Q* = 20 µl s^−1^ whereas for lower or higher flow rates less scattering intensity from native BSA is recordable. For *d*_i_ = 0.9 mm, *I*_int_ is larger for *Q* = 10 µl s^−1^ and 20 µl s^−1^ than for *d*_i_ = 1.7 mm. Only at *Q* = 40 µl s^−1^ is the signal from the larger sample path length higher. More interesting to note is that the optimum conditions are present at *Q* = 10 µl s^−1^ where the highest signal is detectable.

These findings and the difference to the data for lysozyme can be directly understood by looking at the *Q* dependence of *t*_av_ and *t*_tot_ (Fig. 5*c* ▸). In the case of the highest flow rate, no radiation damage is present for both capillaries, all curves can be averaged, and thus the total SAXS intensity is higher for *d*_i_ = 1.7 mm because more proteins give rise to the signal (however, at the cost of a less efficient sample consumption, see §3.1). With decreasing flow rate, radiation damage sets in for the large capillary, leading to the best measured condition for *Q* = 20 µl s^−1^ (longest exposure time *t*_av_). In contrast, for *d*_i_ = 0.9 mm, nearly no radiation damage is present for all conditions studied where the sample is moved. Thus, the sample is in this case ‘under-exposed’ as more frames could have been collected before the onset of the radiation damage.

#### 3.3.4. Influence of the capillary size on the 1D-data noise level   

Ultimately, the goal for the structural bioSAXS experiments is to collect the optimal SAXS pattern (with the lowest noise possible) from a given sample volume, and the noise level for the averaged SAXS curves is discussed in detail below.

**Figure 6:**
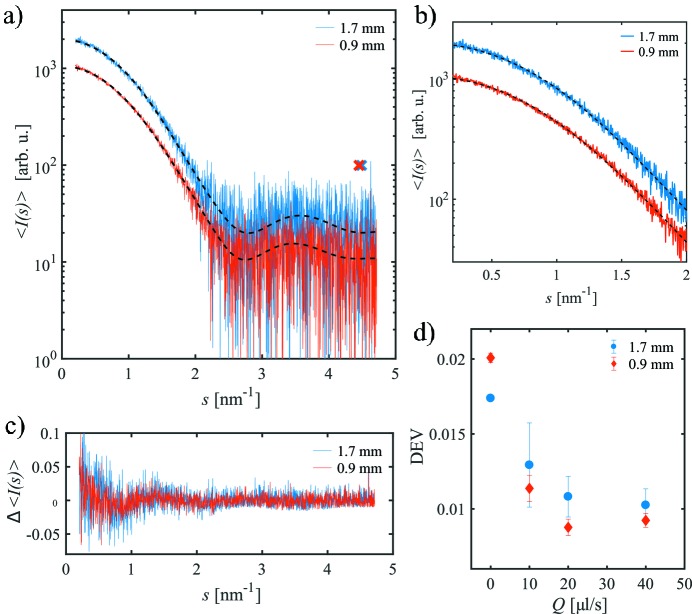
(*a*) Frame-averaged SAXS curves 〈*I*(*s*)〉 for the lysozyme sample in the *d*_i_ = 1.7 mm and *d*_i_ = 0.9 mm capillaries at a total volume flow rate *Q* = 20 µl s^−1^. Dashed lines are the corresponding smooth *AUTOGNOM* curves. The maximum useful data ranges *s*_opt_ are marked by crosses. (*b*) Zoom of (*a*) at smaller *s*. The noise level for the smaller capillary is improved compared with the larger one. (*c*) Normalized deviation from the *AUTOGNOM* curve, Δ〈*I*(*s*)〉, for both curves. (*d*) Parameter DEV as a function of the total volume flow rate *Q* for both capillaries.

Fig. 6(*a*) ▸ shows the frame-averaged SAXS curves 〈*I*(*s*)〉 for the lysozyme sample obtained in both capillaries together with smoothed *AUTOGNOM* curves for *Q* = 20 µl s^−1^. The scattering intensity per frame for the *d*_i_ = 1.7 mm capillary is higher than for the *d*_i_ = 0.9 mm capillary as discussed before (§3.2) but the noise level is changed due to the larger number of frames used for averaging. The noise in the averaged curves (Figs. 6 ▸ *a* and 6*b*) is more clear in the normalized difference between the experimental data and the smoothed curve, Δ〈*I*(*s*)〉 [see equation (2), Fig. 6*c* ▸]. As can be seen, Δ〈*I*(*s*)〉 is fluctuating around zero, whereas the amplitude of these fluctuations is slightly larger for *d*_i_ = 1.7 mm.

To quantify the differences, the parameter DEV has been computed for all 〈*I*(*s*)〉 following equation (3), and the results are shown in Fig. 6(*d*) ▸. For the fixed sample (*Q* = 0 µl s^−1^), DEV is higher in *d*_i_ = 0.9 mm because the radiation damage is already visible after 45 ms in both capillaries and the highest signal is obtained for the optimal path length. As mentioned before, radiation damage present already for the first frames cannot be ruled out and therefore this case is not considered further. For the flowing samples, DEV is smaller for *d*_i_ = 0.9 mm than for *d*_i_ = 1.7 mm, indicating a lower relative noise. The lowest values are reached for *Q* = 20 µl s^−1^ and 40 µl s^−1^, the rates that also yield both similar strong total scattering intensities *I*_int_ and the times *t*_av_ without radiation damage.

The useful data range as well as the optimum number of Shannon channels obtained for the two capillaries at the different flow rates do not show an obvious dependence on the capillary diameter. For fixed samples, the useful *s*-range is limited to *s*_opt_ < 4.0 nm^−1^ and *M*_S_ = 8. When flowing the protein solutions, the scattering intensity at larger angles can be used to determine structural information (*s*_opt_ < 4.5 nm^−1^ and *M*_S_ = 10).

For the more stable BSA samples, which are under-exposed for the *d*_i_ = 0.9 mm capillary, the basic findings are similar (§S4, Fig. S4). Here, the difference between both capillaries is not as pronounced as for the lysozyme samples because of similar strong integrated scattering intensity.

## 4. Conclusion   

We have shown that for highly radiation-sensitive samples, such as biological macromolecules in solution, the use of cylindrical capillaries with path length smaller than the theoretically calculated optimum can yield a more efficient sample consumption and better quality SAXS data under continuous flow of the samples through the X-ray beam. This effect has been demonstrated by comparing two capillary diameters (*d*_i_ = 1.7 mm, close to optimal for 10 keV; *d*_i_ = 0.9 mm, sub-optimal) at various volume flow rates.

The determined SAXS curves for the different settings have been analysed in terms of sample consumption, onset of radiation damage, integrated scattering intensity, and noise level. Based on these parameters, it was found that capillary diameters of *d*_i_ = 0.9 mm yield better SAXS curves for highly radiation-sensitive samples compared with those of *d*_i_ = 1.7 mm. For the smaller diameter the sample consumption is more efficient allowing for more repetitions using a given sample volume. For the same volume flow rates, higher flow speeds are achieved in smaller capillaries, which leads to a faster replenishing of the sample thus diminishing the radiation damage. The larger number of damage-free single SAXS curves and, thus, the overall higher integrated scattering intensity outweighs the effect of the sub-optimal path length. Based on these results, the use of smaller capillaries can be advantageous for standard batch mode measurements, as performed at several bioSAXS beamlines. These findings are confirmed by additional measurements performed at a different SAXS beamline (BM29, ESRF) with different experimental parameters (see §S5).

In contrast to macromolecular crystallography where the radiation damage at cryo-temperatures depends directly on the dose deposited on the sample and can thus be taken into account before the measurement (Garman, 2010 ▸), in bioSAXS the damage strongly depends on the protein’s tendency to form aggregates, which is not necessarily known *a priori* and may also be buffer-dependent. Optimizing the data collection, as shown here, will allow SAXS data to be safely obtained with reduced radiation damage.

Based on the comparison of the data from lysozyme and BSA under different flow rates, two scenarios were found: (i) the sample exhibits significant radiation damage and is thus heavily over-exposed; (ii) the sample does not show large damage and is under-exposed. The optimum condition would be the situation in which as many SAXS curves as possible are collected that are free from radiation damage. A criterion for this could be to compare the exposure time *without* radiation damage, *t*_av_, and the total exposure time, *t*_tot_, for different volume flow rates. Whereas in the case of *d*_i_ = 0.9 mm for lysozyme an optimum condition might be present for slightly lower flow rates than *Q* = 40 µl s^−1^ and increased numbers of SAXS curves, in the case of BSA even slightly lower flow rates than *Q* = 10 µl s^−1^ can yield longer life times. The quantities *t*_av_ and *t*_tot_ as well as *Q* can be easily determined and changed during the SAXS measurement on a given sample to yield the optimum conditions providing the longest in-beam integrity. Alternatively, the sample could be flown at high speed; interactive data analysis would indicate if the sample is damaged. If this is not the case, the measurement could be repeated using the same sample until radiation damage is noticed. This approach is basically not limited to linear flows but might also be used with oscillating flows.

The presented results are of special relevance for the beamlines conducting bioSAXS measurements on solutions, both with and without automatic sample changers. The continuous decrease of X-ray beam sizes into the micrometre regime and the development of more brilliant X-ray sources has spurred the use of microfluidic devices, in particular to study rapid mixing and shearing of protein solutions with limited sample consumption (Brennich *et al.*, 2011 ▸; Graceffa *et al.*, 2013 ▸; Pham *et al.*, 2017 ▸; Schwemmer *et al.*, 2016 ▸; Skou *et al.*, 2014*b* ▸; Wieland *et al.*, 2016 ▸). Due to an even smaller path length down to several tens of micrometres and the strong background contribution from scattering of the wall material, optimized schemes for data collection, like the one proposed here, would be of importance.

Optimal designs for flow-through environments would be highly asymmetric with a thickness of *d*_opt_ and a vertical dimension in the range of the X-ray beam, *i.e.* several micrometres. However, the reproducible fabrication of such a device is a non-trivial task. Moreover, for the selection of the proper material the mechanical stability, vacuum capability, radiation robustness, as well as a low background signal over a large *s*-range have to be taken into account. Using standard cylindrical glass capillaries with a reduced diameter is an easy alternative to other miniaturization approaches. Overall, the use of smaller capillary appears as the most straightforward way to conduct measurements and obtain the high-quality data free from radiation damage.

## 5. Related literature   

The following reference, not citied in the main body of the paper, has been cited in the supporting information: Henke *et al.* (1993 ▸).

## Supplementary Material

Sections S1 to S5; Tables S1 to S2; Figures S1 to S5.. DOI: 10.1107/S1600577518007907/co5096sup1.pdf

## Figures and Tables

**Table 1:** Comparison of the sample volumes used for both capillaries All values were calculated for a total volume *V*_tot_ = 20 µl. *d*_i_: inner capillary diameter; *V*_sample_: volume needed to fill the capillary for full exposure; *V*_beam_: focal volume of the beam, *i.e*. the volume exposed for fixed sample; *V*_exp_: volume exposed for flowing sample; Δ*V*_loss, fix_: loaded volume not exposed to the beam for fixed sample; Δ*V*_loss, flow_: loaded volume not exposed to the beam for flowing sample; *V*_beam_/*V*_sample_: consumption efficiency.

*d*_i_ (mm)	*V*_sample_ (nl)	*V*_beam_ (nl)	*V*_exp_ (µl)	Δ*V*_loss,fix_ (nl)	Δ*V*_loss,flow_ (µl)	*V*_beam_/*V*_sample_ (%)
1.7	794	178	4.47	617	15.53	22
0.9	223	93	8.33	130	11.67	42

**Table 2:** Comparison of the experimental parameters for both capillaries *Q*: total volume flow rate; *v*: linear flow speed; *t*_dwell_: average dwell time in the X-ray beam; Re: Reynolds number (for water); *N*_SAXS_: number of SAXS curves taken; *t*_tot_: total exposure time of the sample. Due to the smaller diameter of the *d*_i_ = 0.9 mm capillary, the flow speeds for equivalent flow rate are higher and the average dwell times shorter.

	*v* (mm s^−1^)		*t*_dwell_ (ms)		Re			
*Q* (µl s^−1^)	1.7 mm	0.9 mm	1.7 mm	0.9 mm	1.7 mm	0.9 mm	*N*_SAXS_	*t*_tot_ (ms)
0	0	0	1000	1000	0	0	20	1000
10	4.4	15.7	45	13	6	11	40	2000
20	8.8	31.4	23	6	11	22	20	1000
40	17.6	62.9	11	3	23	43	10	500

## References

[bb1] Acerbo, A. S., Cook, M. J. & Gillilan, R. E. (2015). *J. Synchrotron Rad.* **22**, 180–186. 10.1107/S1600577514020360 PMC4294029 25537607

[bb2] Blanchet, C. E., Spilotros, A., Schwemmer, F., Graewert, M. A., Kikhney, A., Jeffries, C. M., Franke, D., Mark, D., Zengerle, R., Cipriani, F., Fiedler, S., Roessle, M. & Svergun, D. I. (2015). *J. Appl. Cryst.* **48**, 431–443. 10.1107/S160057671500254X PMC4379436 25844078

[bb3] Brennich, M. E., Nolting, J. F., Dammann, C., Nöding, B., Bauch, S., Herrmann, H., Pfohl, T. & Köster, S. (2011). *Lab Chip*, **11**, 708–716. 10.1039/c0lc00319k 21212871

[bb4] Brooks-Bartlett, J. C., Batters, R. A., Bury, C. S., Lowe, E. D., Ginn, H. M., Round, A. & Garman, E. F. (2017). *J. Synchrotron Rad.* **24**, 63–72. 10.1107/S1600577516015083 PMC5182020 28009547

[bb5] Classen, S., Hura, G. L., Holton, J. M., Rambo, R. P., Rodic, I., McGuire, P. J., Dyer, K., Hammel, M., Meigs, G., Frankel, K. A. & Tainer, J. A. (2013). *J. Appl. Cryst.* **46**, 1–13. 10.1107/S0021889812048698 PMC3547225 23396808

[bb6] David, G. & Pérez, J. (2009). *J. Appl. Cryst.* **42**, 892–900.

[bb7] Fischetti, R. F., Rodi, D. J., Mirza, A., Irving, T. C., Kondrashkina, E. & Makowski, L. (2003). *J. Synchrotron Rad.* **10**, 398–404. 10.1107/s0909049503016583 12944630

[bb8] Franke, D., Jeffries, C. M. & Svergun, D. I. (2015). *Nat. Methods*, **12**, 419–422. 10.1038/nmeth.3358 25849637

[bb9] Franke, D., Kikhney, A. G. & Svergun, D. I. (2012). *Nucl. Instrum. Methods Phys. Res. A*, **689**, 52–59.

[bb10] Franke, D., Petoukhov, M. V., Konarev, P. V., Panjkovich, A., Tuukkanen, A., Mertens, H. D. T., Kikhney, A. G., Hajizadeh, N. R., Franklin, J. M., Jeffries, C. M. & Svergun, D. I. (2017). *J. Appl. Cryst.* **50**, 1212–1225. 10.1107/S1600576717007786 PMC5541357 28808438

[bb11] Garman, E. F. (2010). *Acta Cryst.* D**66**, 339–351. 10.1107/S0907444910008656 PMC2852297 20382986

[bb12] Garrison, W. M. (1987). *Chem. Rev.* **87**, 381–398.

[bb13] Gasteiger, E., Hoogland, C., Gattiker, A., Duvaud, S., Wilkins, M. R., Appel, R. D. & Bairoch, A. (2005). *Protein Identification and Analysis Tools on the ExPASy Server, in The Proteomics Protocols Handbook*, edited by J. M. Walker, pp. 571–607. Totowa: Humana Press.

[bb14] Graceffa, R., Nobrega, R. P., Barrea, R. A., Kathuria, S. V., Chakravarthy, S., Bilsel, O. & Irving, T. C. (2013). *J. Synchrotron Rad.* **20**, 820–825. 10.1107/S0909049513021833 PMC3795536 24121320

[bb15] Graewert, M. A. & Svergun, D. I. (2013). *Curr. Opin. Struct. Biol.* **23**, 748–754. 10.1016/j.sbi.2013.06.007 23835228

[bb16] Guinier, A. (1939). *Ann. Phys.* **11**, 161–237.

[bb17] Hajizadeh, N. R., Franke, D. & Svergun, D. I. (2018). *J. Synchrotron Rad.* **25**, 906–914. 10.1107/S1600577518005398 PMC5929361 29714204

[bb18] Henke, B. L., Gullikson, E. M. & Davis, J. C. (1993). *At. Data Nucl. Data Tables*, **54**, 181–342.

[bb21] Hopkins, J. B. & Thorne, R. E. (2016). *J. Appl. Cryst.* **49**, 880–890. 10.1107/S1600576716005136 PMC4886981 27275138

[bb22] Inoue, K., Doutch, J. & Terrill, N. (2013). *Bunseki Kagaku*, **62**, 565–570.

[bb23] Jeffries, C. M., Graewert, M. A., Blanchet, C. E., Langley, D. B., Whitten, A. E. & Svergun, D. I. (2016). *Nat. Protoc.* **11**, 2122–2153. 10.1038/nprot.2016.113 PMC5402874 27711050

[bb24] Jeffries, C. M., Graewert, M. A., Svergun, D. I. & Blanchet, C. E. (2015). *J. Synchrotron Rad.* **22**, 273–279. 10.1107/S1600577515000375 25723929

[bb26] Kirby, N., Cowieson, N., Hawley, A. M., Mudie, S. T., McGillivray, D. J., Kusel, M., Samardzic-Boban, V. & Ryan, T. M. (2016). *Acta Cryst.* D**72**, 1254–1266. 10.1107/S2059798316017174 PMC5137223 27917826

[bb27] Kirby, N. M., Mudie, S. T., Hawley, A. M., Cookson, D. J., Mertens, H. D. T., Cowieson, N. & Samardzic-Boban, V. (2013). *J. Appl. Cryst.* **46**, 1670–1680.

[bb28] Konarev, P. V. & Svergun, D. I. (2015). *IUCrJ*, **2**, 352–360. 10.1107/S2052252515005163 PMC4420545 25995844

[bb29] Kuwamoto, S., Akiyama, S. & Fujisawa, T. (2004). *J. Synchrotron Rad.* **11**, 462–468. 10.1107/S0909049504019272 15496733

[bb30] Li, N., Li, X., Wang, Y., Liu, G., Zhou, P., Wu, H., Hong, C., Bian, F. & Zhang, R. (2016). *J. Appl. Cryst.* **49**, 1428–1432. 10.1107/S160057671601195X PMC5045727 27738413

[bb31] Maleknia, S. D., Ralston, C. Y., Brenowitz, M. D., Downard, K. M. & Chance, M. R. (2001). *Anal. Biochem.* **289**, 103–115. 10.1006/abio.2000.4910 11161303

[bb32] Meisburger, S. P., Warkentin, M., Chen, H., Hopkins, J. B., Gillilan, R. E., Pollack, L. & Thorne, R. E. (2013). *Biophys. J.* **104**, 227–236. 10.1016/j.bpj.2012.11.3817 PMC3540250 23332075

[bb33] Nielsen, S. S., Møller, M. & Gillilan, R. E. (2012). *J. Appl. Cryst.* **45**, 213–223. 10.1107/S0021889812000957 PMC3325496 22509071

[bb34] Pernot, P., Round, A., Barrett, R., De Maria Antolinos, A., Gobbo, A., Gordon, E., Huet, J., Kieffer, J., Lentini, M., Mattenet, M., Morawe, C., Mueller-Dieckmann, C., Ohlsson, S., Schmid, W., Surr, J., Theveneau, P., Zerrad, L. & McSweeney, S. (2013). *J. Synchrotron Rad.* **20**, 660–664. 10.1107/S0909049513010431 PMC3943554 23765312

[bb36] Petoukhov, M. V., Franke, D., Shkumatov, A. V., Tria, G., Kikhney, A. G., Gajda, M., Gorba, C., Mertens, H. D. T., Konarev, P. V. & Svergun, D. I. (2012). *J. Appl. Cryst.* **45**, 342–350. 10.1107/S0021889812007662 PMC4233345 25484842

[bb37] Pham, N., Radajewski, D., Round, A., Brennich, M., Pernot, P., Biscans, B., Bonneté, F. & Teychené, S. (2017). *Anal. Chem.* **89**, 2282–2287. 10.1021/acs.analchem.6b03492 28192906

[bb39] Rogers, D. F. (1992). *Laminar Flow Analysis.* Cambridge University Press.

[bb40] Rott, N. (1990). *Annu. Rev. Fluid Mech.* **22**, 1–12.

[bb41] Round, A., Felisaz, F., Fodinger, L., Gobbo, A., Huet, J., Villard, C., Blanchet, C. E., Pernot, P., McSweeney, S., Roessle, M., Svergun, D. I. & Cipriani, F. (2015). *Acta Cryst.* D**71**, 67–75. 10.1107/S1399004714026959 PMC4304687 25615861

[bb43] Sahle, C. J., Henriquet, C., Schroer, M. A., Juurinen, I., Niskanen, J. & Krisch, M. (2015). *J. Synchrotron Rad.* **22**, 1555–1558. 10.1107/S1600577515016331 26524322

[bb44] Schroer, M. A. & Svergun, D. I. (2018). *Emerg. Top. Life Sci.* **2**, 69–79. 10.1042/ETLS20170138 33525782

[bb45] Schwemmer, F., Blanchet, C. E., Spilotros, A., Kosse, D., Zehnle, S., Mertens, H. D. T., Graewert, M. A., Rössle, M., Paust, N., Svergun, D. I., von Stetten, F., Zengerle, R. & Mark, D. (2016). *Lab Chip*, **16**, 1161–1170. 10.1039/c5lc01580d 26931639

[bb47] Skou, M., Skou, S., Jensen, T. G., Vestergaard, B. & Gillilan, R. E. (2014*b*). *J. Appl. Cryst.* **47**, 1355–1366. 10.1107/S1600576714012618 PMC4119951 25242913

[bb46] Skou, S., Gillilan, R. E. & Ando, N. (2014*a*). *Nat. Protoc.* **9**, 1727–1739. 10.1038/nprot.2014.116 PMC4472361 24967622

[bb48] Svergun, D. I. & Koch, M. H. J. (2003). *Rep. Prog. Phys.* **66**, 1735–1782.

[bb49] Svergun, D. I., Koch, M. H. J., Timmins, P. A. & May, R. P. (2013). *Small Angle X-ray and Neutron Scattering from Solutions of Biological Macromolecules.* Oxford University Press.

[bb50] Wieland, D. C. F., Garamus, V. M., Zander, T., Krywka, C., Wang, M., Dedinaite, A., Claesson, P. M. & Willumeit-Rö*mer*, R. (2016). *J. Synchrotron Rad.* **23**, 480–486. 10.1107/S1600577515024856 26917136

