# Supplementary material for: Smaller capillaries improve the small-angle X-ray scattering signal and sample consumption for biomacromolecular solutions
Source: J Synchrotron Radiat. 2018 Jun 26;25(Pt 4):1113–22. doi: 10.1107/S1600577518007907 (PMC6038601; doi:10.1107/S1600577518007907)
Supplement: Supplementary file 1 [file s-25-01113-sup1.pdf]

JOURNAL OF SYNCHROTRON RADIATION

Volume 25 (2018)

Supporting information for article:

Smaller capillaries improve the small-angle X-ray scattering signal and sample consumption for biomacromolecular solutions

Martin A. Schroer, Clement E. Blanchet, Andrey Yu. Gruzinov, Melissa A. Gräwert, Martha E. Brennich, Nelly R. Hajizadeh, Cy M. Jeffries and Dmitri I. Svergun

S1. Optimal path length for scattering at different photon energies

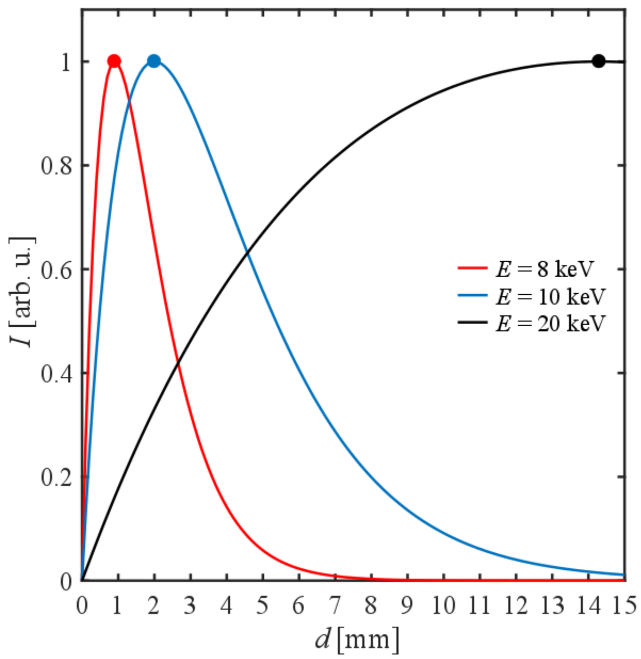

Figure S1 The relative magnitude of the total scattering intensity, *I*, calculated from pure water as a function of sample path length *d* at three different X-ray energies, *E*. The maximum calculated scattering intensity shifts to longer path lengths as the energy increases (the maxima d_{opt} are marked by dots). The plots are normalized on the maximum intensity (energy dependent total linear attenuation coefficient is taken from Henke *et al.*, 1993).

S2. Radiation dose for sample flow

Following the approach of Jeffries *et al.*, 2015, and Hopkins & Thorne, 2016, the radiation dose D on the samples is calculated as

$$D = 1000 \frac{F\,E\,t}{A_{Xray}\,\rho_m\,d}[1 - \exp(-\mu d)].$$

Here, F is the beam flux (photons s^{-1}), E the photon energy (J $photon^{-1}$), t the time the same sample spot is exposed, A_{Xray} the beam profile, ρ_m the mass density of the sample (g cm^{-3}), d the path length and μ the linear absorption coefficient. The factor 1000 converts J g^{-1} to J kg^{-1} to obtain Gy.

The path length is given by the respective capillary diameters. For linear capillary flow, the time the same sample is actually exposed to the beam is given by the average dwell time t_{dwell}. The parameters present at P12 are discussed in §2.2 (assuming a Gaussian beam profile)). The values of ρ_m and μ for the protein solutions studied are taken from Jeffries *et al.*, 2015, which studied similar protein solutions (lysozyme: ρ_m= 1.028 g cm^{-3}, μ = 5.56 cm^{-1}; BSA: ρ_m= 1.023 g cm^{-3}, μ = 5.27 cm^{-1}). The transmission T_{SiO2} of quartz glass wall of the capillaries has to be taken into account in addition (d_{i} = 1.7 mm: T_{SiO2} = 0.81; d_{i} = 0.9 mm: T_{SiO2} = 0.79). The so computed average radiation doses are given in Table S1 and Table S2.

For non-flowing conditions, the average dose is higher for the smaller capillary, whereas with flow the dose is lower than for the larger one.

Table S1 Average radiation dose for lysozyme

Q [µl/s]	1.7 mm: t_{dwell} [ms]	0.9 mm: t_{dwell} [ms]	1.7 mm: D [kGy]	0.9 mm: D [kGy]
0	1000	1000	13.96	17.34
10	45	13	0.63	0.23
20	23	6	0.32	0.10
40	11	3	0.15	0.05

Table S2 Average radiation dose for BSA

Q [µl/s]	1.7 mm: t_{dwell} [ms]	0.9 mm: t_{dwell} [ms]	1.7 mm: D [kGy]	0.9 mm: D [kGy]
0	1000	1000	13.6	16.73
10	45	13	0.61	0.22
20	23	6	0.31	0.10
40	11	3	0.15	0.05

S3. Radial velocity profiles

The radial velocity profiles of a liquid in the linear flow regime can be approximated as (Rogers, 1992)

$$v_{\mathrm{r}}(r) = 2\,v\left(1 - \frac{r^2}{R^2}\right)$$

Herein v is the linear flow speed, r the radial distance from the center of the capillary and $R = 0.5\ d_{\mathrm{i}}$ the capillary radius. At the border of the capillary ($r = R$) the velocity approaches zeros. For the d_{i} = 0.9 mm capillary much higher flow velocities are reached over a large part of the profile, which results in a stronger exchange of sample. Only at the lowest flow rate measured at (Q = 10 µl/min), the velocity profile for the d_{i} = 0.9 mm capillary is smaller than at for the d_{i} = 1.7 mm capillary at the highest flow rate.

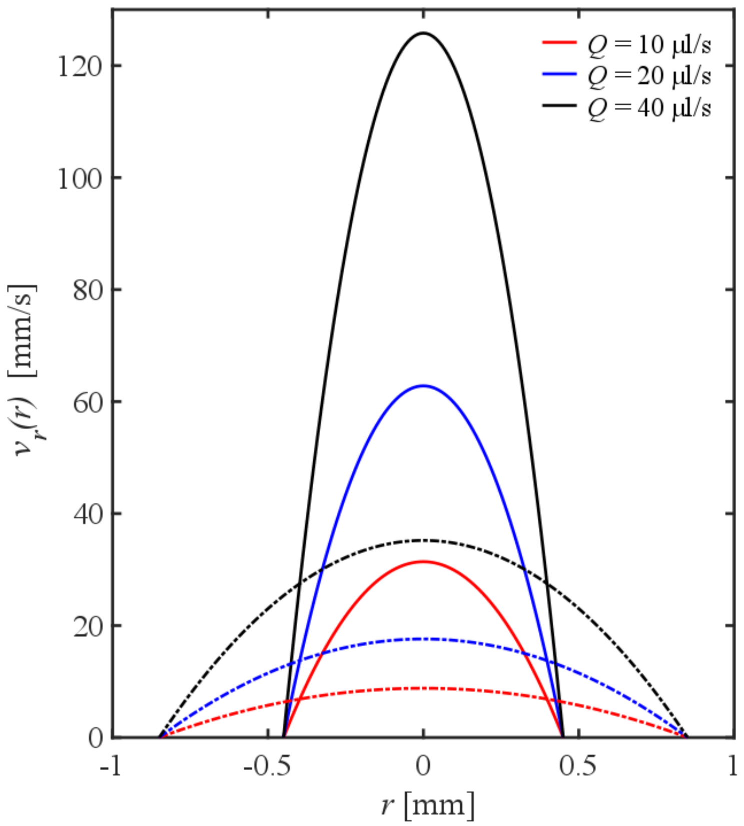

Figure S2 Radial velocity profiles for the d_{i} = 0.9 mm (solid lines) and the d_{i} = 1.7 mm (dashed dotted lines) capillaries at different volume flow rates.

S4. Results for BSA

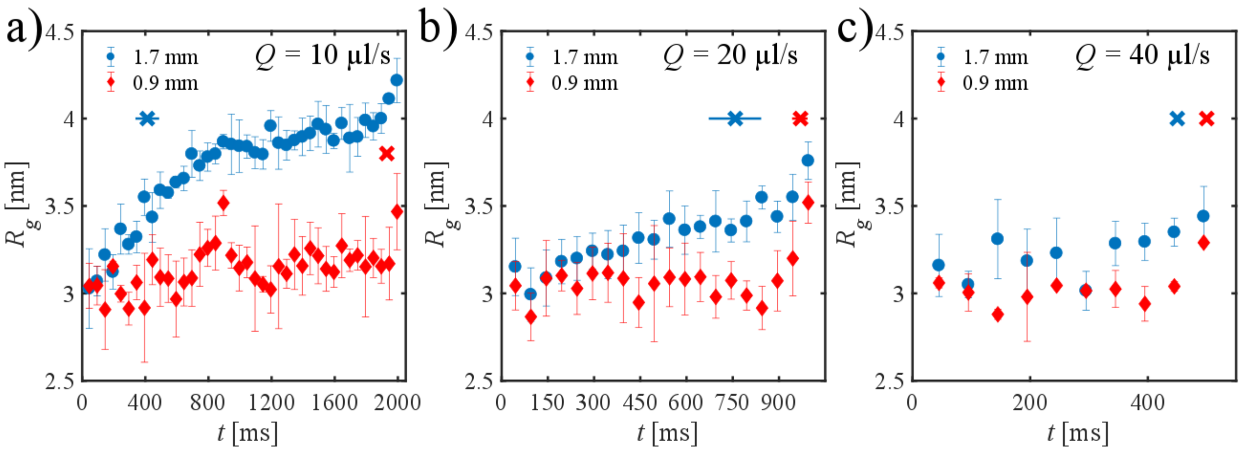

Figure S3 Radius of gyration, R_g, of BSA as a function of exposure time t at different flow rates for both capillaries: a) Q = 10 µl/s, b) Q = 20 µl/s, c) Q = 40 µl/s. The crosses mark the number of single SAXS curves accepted by *CorMap*. (Blue: 1.7 mm; red: 0.9 mm). For the BSA samples, the onset of radiation-induced changes starts at longer exposure time for d_i = 0.9 mm than for d_i = 1.7 mm (see *CorMap* results). Especially for the flow rates studied here, nearly all SAXS curves are similar for the smaller capillary. In this situation, the BSA solutions are even partially under-exposed, allowing to collect more SAXS curves before radiation damage sets in and thus yielding lower noise data.

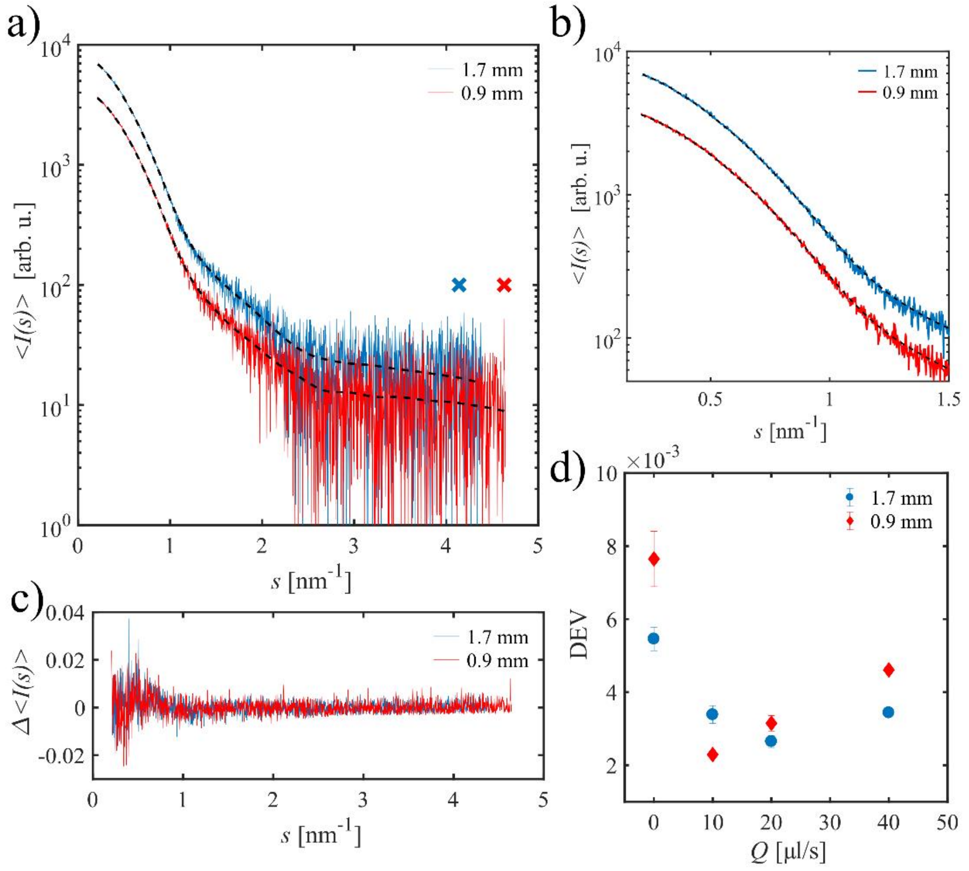

Figure S4 Averaged SAXS curves *<I(s)>* for the BSA sample in the d_i = 1.7 mm and d_i = 0.9 mm capillaries at a total volume flow rate Q = 20 µl/s. Dashed lines are the corresponding *AUTOGNOM* curves. The maximum useful data ranges s_{opt} are marked by crosses. b) Zoom of a) at smaller s. c) Normalized deviation from the *AUTOGNOM* curves, Δ*<I(s)>*, for both curves. d) Parameter DEV as a function of the total volume flow rate Q for both capillaries.

S5. Results from beamline BM29

Additional SAXS measurements on both type of capillaries have been performed at beamline BM29, ESRF, Grenoble (Pernot *et al.*, 2015), using at photon energy of E = 12.5 keV (λ = 0.099 nm) with a beam size of 700 µm x 700 µm (v x h, full width half maximum, FWHM) and a flux of 1.4 x 10^{12} photons s^{-1} at a storage ring current of 200 mA. For this X-ray energy, the optimum path length for water is d_{opt} = 3.9 mm. Thus, the diameter for both capillaries are much smaller than $d_{opt.}$ Similar to the measurements at P12, standard batch mode measurements were performed using a robotic sample changer (Round *et al.*, 2015) both with continuous in-capillary sample flow through the beam line under vacuum. At BM29, a constant volume of V_{tot} = 30 µl was loaded into the capillaries that were maintained at a constant temperature of T = 20 °C.

SAXS data were recorded from lysozyme solutions (concentrations: 2.5 mg/mL; 4.8 mg/mL; 9.7 mg/mL) in a similar buffer as used for the P12 measurements. Data were collected with a PILATUS 1M photon counting detector (DECTRIS, Switzerland) from samples and buffers at several different flow rates and total exposure times. For each run, ten 2D-SAXS data were recorded at different collection times per frame from 0.5 s to 3.6 s resulting in different flow rates (Q = 2 – 6 µl/s). (Here, the flow rate Q was given as $Q = V_{tot} / (t_{exp} * 10$ frames + 10 s)).

For the averaged SAXS curves, the exposure time without radiation damage, t_{av}, as well as the DEV parameter were determined as described in the main text (Fig. S5). In case of the d_i = 0.9 mm capillary, radiation damage sets in latter than for d_i = 1.7 mm. The resulting averaged SAXS curves therefore have a lower DEV parameter in case of the smaller capillary. Based on the proposed criterion, the best flow rate is present for $Q \approx 3$ µl/s.

The data obtained from BM29 using different experimental parameters thus confirm that in case of radiation-damage sensitive samples, the use of capillaries with smaller diameters can yield SAXS curves of higher quality.

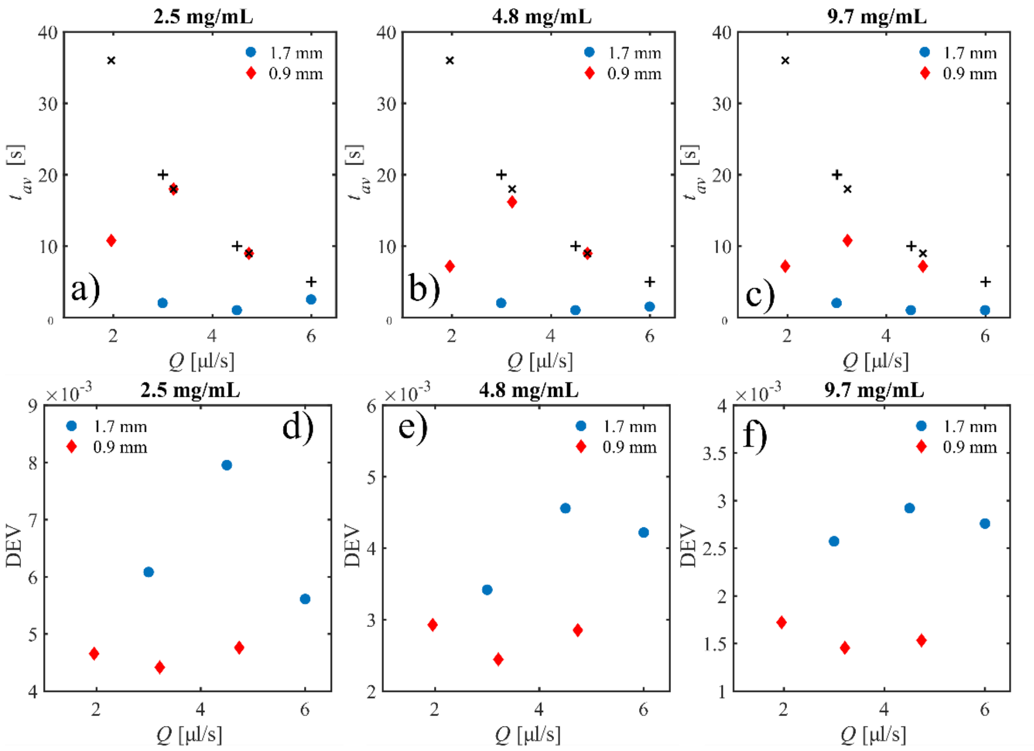

Figure S5 Exposure time before showing radiation damage, t_{av}, (a)-c)) as well as the DEV parameter (d)-f)) obtained for lysozyme solutions of different concentrations and flow rate for the two capillaries. Crosses in a)-c) mark the total exposure time.

References

Henke, B. L., Gullikson, E. M. & Davis, J. C. (1993). *At. Data Nuc. Data Tables* **54**, 181-342.

Hopkins, J. B. & Thorne, R. E. (2016). *J. Appl. Cryst.* **49**, 880-890.

Jeffries, C. M., Graewert, M. A., Svergun, D. I. & Blanchet, C. E. (2015). *J. Synchrotron Rad.* **22**, 273-279.

Pernot, P., Round, A., Barrett, R., De Maria Antolinos, A., Gobbo, A., Gordon, E., Huet, J., Kieffer, J., Lentini, M., Mattenet, M., Morawe, C., Mueller-Dieckmann, C., Ohlsson, S., Schmid, W., Surr, J., Theveneau, P., Zerrad, L. & McSweeney, S. (2013). *J. Synchrotron Rad.* **20**, 660-664.

Round, A., Felisaz, F., Fodinger, L., Gobbo, A., Huet, J., Villard, C., Blanchet, C. E., Pernot, P., McSweeney, S., Roessle, M., Svergun, D. I. & Cipriani, F. (2015). *Acta Cryst.* D**71**, 67-75.

Rogers, D. F. (1992). *Laminar Flow Analysis*. Cambridge University Press.
